# Deprivation of human natural killer cells and antitumor immune response

**DOI:** 10.5195/cajgh.2013.98

**Published:** 2014-01-24

**Authors:** Vyacheslav Ogay, Aliya Sekenova, Inpyo Choi

**Affiliations:** 1 Laboratory of Stem Cells, National Center for Biotechnology, Almaty, Kazakhstan; 2 Immunotherapy Research Center, Korea Research Institute of Bioscience and Biotechnology, Daejeon, Republic of Korea

**Keywords:** human natural killer cells, antitumor immune response, oncologic diseases

## Abstract

**Introduction:**

Cell-based immunotherapy has been given increased attention as a treatment for cancer. Human natural killer (NK) cells are resident lymphocyte populations. They exhibit potent antitumor activity without human leukocyte antigen matching and without prior antigen exposure. They also are a promising tool for immunotherapy of solid and hematologic cancers. However, most cancer patients do not have enough NK cells to induce an effective antitumor immune response. This demonstrates a need for a source of NK cells that can supplement the endogenous cell population.

**Material and methods:**

In this study, we derived induced pluripotent stem cells (iPSCs) from peripheral blood T-lymphocytes using Sendai virus vectors.

**Results:**

Generated iPSCs exhibited monoclonal T cell receptors (TCR) rearrangement in their genome, a hallmark of mature terminally differentiated T cells. These iPSCs were differentiated into NK cells using a two-stage coculture system: iPSCs into hematopoietic CD34+ cells with feeder cells M210-B4 (ATCC, USA) and CD34+ cells into mature NK cells with AFT024 cells (ATCC, USA). Our results showed that iPSC-derived NK cells expressed CD56, CD16, NKp 44 and NKp 46, possessed high cytotoxic activity and produced high level of interferon-γ.

**Conclusion:**

Based on our data, derivation of NK cells from induced pluripotent stem cells should be considered in the treatment of oncologic diseases. This would allow for the development of cell therapy for cancer using immunologically compatible NK cells derived from iPSCs. This may contribute to a more efficient treatment of oncologic diseases in addition to traditional cancer treatment.

